# Retraction Note: hERG1 channels modulate integrin signaling to trigger angiogenesis and tumor progression in colorectal cancer

**DOI:** 10.1038/s41598-025-87746-6

**Published:** 2025-01-28

**Authors:** Olivia Crociani, Francesca Zanieri, Serena Pillozzi, Elena Lastraioli, Matteo Stefanini, Antonella Fiore, Angelo Fortunato, Massimo D’Amico, Marika Masselli, Emanuele De Lorenzo, Luca Gasparoli, Martina Chiu, Ovidio Bussolati, Andrea Becchetti, Annarosa Arcangeli

**Affiliations:** 1 Department of Experimental and Clinical Medicine, Section of Internal Medicine, University of Florence and Istituto Toscano Tumori (ITT), Florence, Italy; 2 Unit of General Pathology Department of Biomedical, Biotechnological and Translational Sciences (SBiBiT), University of Parma, Parma, Italy; 3 Department of Biotechnology and Biosciences, University of Milano Bicocca, Milano, Italy

Retraction of: *Scientific Reports* 10.1038/srep03308, published online 25 November 2013

The Editors have retracted this Article.

After the publication of this Article, concerns were raised regarding the high similarity between some bands in the α-Tubulin blot of Figure 4A and the α-Tubulin and α-total S6K1 blots of Figure 4B. An Investigation by the Editors has uncovered further issues such as other similarities between different blots (e.g. left α-Akt blot of Figure 2D and α-p-GSK-3 blot of Figure S3; HCT116 and HCT8 α-p85 blots of Figure 2C) and inaccurate molecular weight labelling (e.g. α-β1 blots of Figures 1B and 1D). Additionally, some original blots provided in the Supplementary Information file do not correspond with cropped blots presented in the main figures (e.g. α-total S6K1 and α-total 4E-BP1 of Figure 4B; Figure 6E).

The Authors were not able to sufficiently explain the discrepancies, and the Editors therefore no longer have confidence in the reliability of the data presented in the Article.

Annarosa Arcangeli has not explicitly stated whether they agree to this retraction. None of the other Authors have responded to any correspondence from the Editors about this retraction. The Editors have not been able to obtain current email addresses for Luca Gasparoli, Marika Masselli and Massimo D’Amico.

